# Co-amoxiclav Effects on the Structural and Binding Properties of Human Serum Albumin

**Published:** 2010

**Authors:** Saeed Hesami Takallu, Mostafa Rezaei Tavirani, Shiva Kalantari, Mahrooz Amir Bakhtiarvand, Sayed Mohammad Mahdavi

**Affiliations:** a *Science & Research Branch, Islamic Azad University (IAU), Tehran, Iran.*; b * Proteomics Research Center, Faculty of Paramedical Sciences, Shaheed Beheshti University of Medical Sciences, Tehran, Iran.*; c *Skin Research Center, Shaheed Beheshti University of Medical Sciences, Tehran, Iran. *

**Keywords:** Human serum albumin, Co-amoxiclav, Charge surface density, Fever, Hexadecyl pyridinium bromide

## Abstract

Human serum albumin (HSA) is the most abundant plasma protein in the human body. HSA plays an important role in drug transport and metabolism. This protein has a high affinity to a very wide range of materials, including metals such as Cu2+ and Zn2+, fatty acids, amino acids and metabolites such as bilirubin and many drug compounds. In this study, we investigated the effects of co-amoxiclav, as a drug which could be carried by this protein, on HSA structure and binding properties via spectroscopy and electrochemistry techniques. Based on this study, it was found that a therapeutic dose of co-amoxiclav as well as doses 4 to 8 folds higher than the therapeutic dose has no considerable effect on the HSA tertiary structure at 37^o^C. However, a dose 2 folds that of the therapeutic dose has a slight effect, but higher doses of the drug has a mild effect in pathological temperature (42^o^C). In addition, charge density of HSA surface is decreased at 42^o^C, compared to 37^o^C. Hence, this finding suggests a reduced role of HSA in regulation of osmotic pressure in the fever conditions, compared to the physiological conditions. Co-amoxiclav reduces the charge surface density of HSA at physiological and pathological temperatures and therefore alters its binding properties, which could be important in drug interference and complications.

## Introduction

Human serum albumin (HSA) is the most abundant plasma protein in the human body, with a plasma concentration of 0.6 mM. HSA plays an important role in drug transport and metabolism. HSA is a 67 kDa single chain, non- glycosylated polypeptide, that folds into a heart-shaped structure containing approximately 67% *α*-helix (1-2). Like most of the plasma proteins, albumin is synthesized in the liver where it is produced at a rate of approximately 0.7 mg/h for every gram of liver (i.e. 10–15 g daily). Human serum albumin exhibits an average half-life of 19 days. The functions and binding properties of HSA are multifold (3): a) it acts as the solubilizing agent for long chain fatty acids and is therefore essential for the metabolism of lipids; b) it binds to bilirubin, the breakdown product of heme; c) it binds to a great number of therapeutic drugs such as penicillins, sulfonamides, indole compounds, and benzodiazepines; d) it binds to copper (II) and nickel (II) in a specific and calcium (II) and zinc (II) in a relatively non-specific manner and acts as the transport vehicle for these metal ions in the blood; e) it is the major protein responsible for the colloidal osmotic pressure of the blood; and f) when HSA is broken down, the amino acids provide nutrition to peripheral tissue (4). It is also responsible for the maintenance of blood pH, the drug disposition and efficacy, and the contribution of colloidal osmotic blood pressure (5).

Enzymatic activity of HSA on different substrates or drugs has been studied and documented. The structural mechanism of this activity is unknown (6). Its physiological and pharmacological properties have been extensively studied over several decades (3, 7, 8). In addition to its ordinary clinical applications, such as hypovolemic shock treatment, many investigators have attempted to utilize HSA as a carrier to deliver various drugs to their specific targets (9-11). For many drugs, binding to serum albumin is a critical determinant of their distribution and pharmacokinetics. However, there has as yet been no high resolution crystal structures published of drug-albumin complexes (13). The most important binding sites on HSA are sites I and II, which are also called warfarin binding sites and benzodiazepine binding sites (13). The principal regions of ligand binding sites of albumin are located in hydrophobic cavities in subdomains IIA and IIIA, which exhibit similar chemistry. 

The IIIA subdomain is the most active in accommodating many ligands, for example, digoxin, ibuprofen, and tryptophan. The distribution of hydrophobic and hydrophilic residues in the binding crevice is distinctly asymmetric. The principal non-polar residues are sequestered into the hydrophobic cavity inside the protein core and the polar residues onto the surface (14).

Co-amoxiclav is a drug consisting of amoxicillin and clavolonic acid. The proprietary combination of amoxicillin and clavolanate potassium provides the double advantages of a broad-spectrum antibiotic activity against many gram-negative and gram-positive organisms and a β-lactamase inhibitor that extends the spectrum of amoxicillin to cover β-lactamase-producing pathogens. This second property of amoxicillin–clavolanate has become increasingly important, as respiratory pathogens (15- 16).

Here we studied the co-amoxiclav effect on the optical properties of HSA, using the uv-vis spectroscopy technique, in order to investigate changes in tertiary structure of HSA at physiological and pathological temperatures in the presence and absence of different doses of drug. The binding properties of the drug have also been studied by the electrochemistry technique, under the same conditions and in the presence of one dose of drug.

## Experimental

*Material*

Human serum albumin (HSA) was purchased form Sigma Chemical Company (USA). Co-amoxiclav was purchased from Glaxo (UK) and the other reagents (Hexa decyl pyridinium bromide, tris base and HCl) were provided from Merck Chemical Company (Germany). Tris buffer 50 mM, pH 7.5, was used for all the spectroscopic experiments.

*UV-visible spectroscopy studies*

The uv-visible spectra of HSA were obtained, using a *Unico *spectrophotometer. The Spectra from 240 nm to 300 nm were drawn in different concentrations of co-amoxiclav. HSA was incubated for 5 min in each concentration of drug in tris buffer 50 mM, under the physiological and pathological temperatures.

The uv-visible spectra were used to study the changes in tertiary structure of the protein. Absorbance in the 280 nm was obtained and illustrated against the drug doses. Incubation of free HSA for 5 min, in the presence and absence of co-amoxiclav does not alter the spectra. Periods of 5 min incubation did not alter the spectra, since the protein samples used in each experiment for all doses were fresh. The protein samples used in each dose were separated and unique, and fresh protein samples were used in each incubation. Hence, the 5 min incubation periods were not added to each other. These separate experiments were done to decrease the probability of heat induced HSA denaturation or conformational changes instead of co-amoxiclav effects. 

*Ligand binding studies*

The ligand binding of HSA-HPB (Hexadecyl pyridinium bromide) interaction at physiological and pathological temperatures and also in the presence of co-amoxiclav was studied, using a Fluke potentiometer. Electrochemical studies were carried out in a conventional three electrode cell, equipped with a Teflon stopper with holes to hold electrodes in appropriate configurations for minimizing the solution resistance in the electrochemical study. Electrochemical impedance measurements were performed at open circuit potential with proper redox potential as bias potential (17). Electrochemical signals were calibrated to HPB concentration, which was used in order to determine the concentration of free HPB. The ratio for HPB binding to each mole of macromolecule were calculated. HSA concentration was 0.4 mg/mL. According to the literature, the therapeutical dosage of co-amoxiclav is 500/125 mg for amoxicillin and clavolonic acid, respectively. Application of the same concentration in this study provided C_max_ of about 10 μg/mL for amoxicillinand 2-4 μg/mL for clavolanate in the plasma.

## Results and Discussion

**Figure 1:**
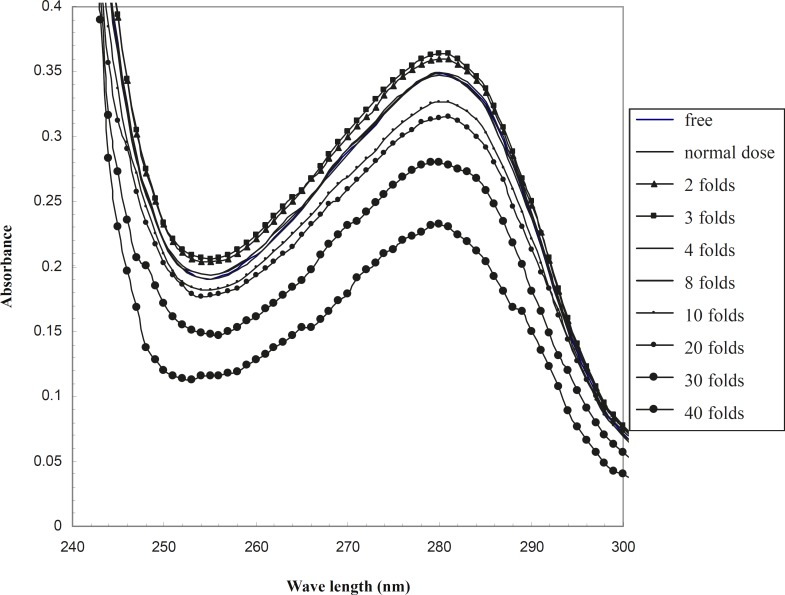
UV-visible spectrum of HSA in the presence of different doses of co-amoxiclav in the phosphate buffer 50 mM, pH 7.5, at 37^o^C

UV-visible spectrum of HSA in the absence and presence of therapeutical and 2-40 folds of therapeutical doses of co-amoxiclav are drawn in order to analyze the tertiary structure of serum albumin in the range of 240 - 300 nm (Figure 1). This wavelength range has been used in the study of HSA structure and conformation, with different methods such as uv-visible spectroscopy, Circular Dichroism and differential scanning Calorimetery in previous studies (19, 20).

Albumin is an apo-protein and its sharp peak at 280 nm relates to the aromatic amino acids, as illustrated in Figure 1. According to this figure, the spectrum of the therapeutical dose of co-amoxiclav at 37^o^C completely corresponds to the spectrum of free drug HSA. Therefore, it could be suggested that optical properties of HSA is not altered in the presence of drug. Co-amoxiclav showed no effect on the tertiary structure of HSA. Studying the structure of HSA by just one method (uv-visible spectroscopy) is questionable. Nevertheless, in this study the change in HSA -HPB binding pattern was appropriate to its structural alteration shown with uv-visible spectroscopy. HSA binding to HPB in the presence or absence of co-amoxiclav shows two distinct patterns. This difference confirms the reliability of uv-visible spectroscopy for these types of studies. 

Raising the concentration of drug up to 2 folds that of the therapeutical dose, affects the tertiary structure and changes HSA spectrum in comparison to the drug free HSA (this effect is very mild). However, a further raise in the concentration of drug to 4 and 8 folds that of the therapeutical dose of co-amoxiclav does not change the spectrum of drug-free HSA. The effect of higher doses of drug on the tertiary structure of protein could be due to protein denaturation or alteration of steriostructure. This effect of drug has been shown in Figure 1. 

In order to compare the effect of co-amoxiclav on the HSA at 37^o^C and 42^o^C, the uv-visible spectra were drawn under the same conditions at pathological temperature. 

**Figure 2:**
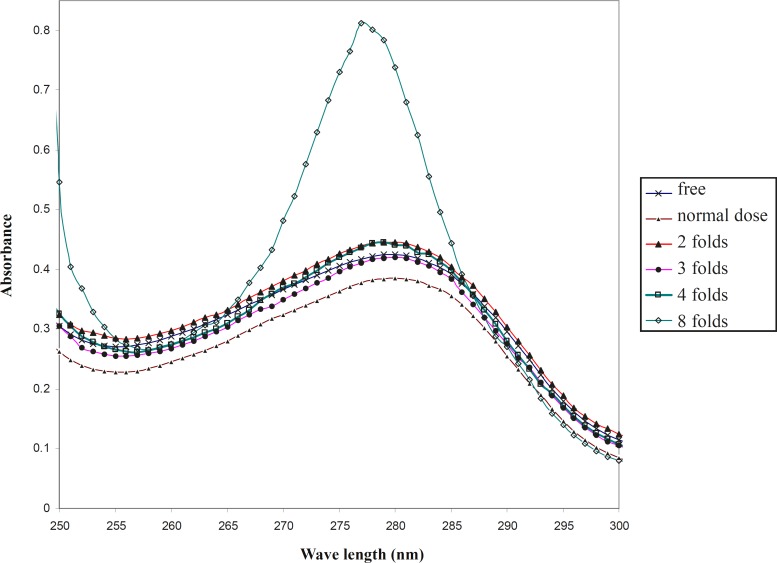
UV-visible spectra of HSA in the presence of different doses of co-amoxiclav in the phosphate buffer 50 mM, pH 7.5 at 42^o^C

In contrast to the physiological temperature, as shown in Figure 2, the optical properties of drug-free HSA is distinct from its properties in the presence of a normal dose of drug at 4^o^C. It could be proposed that co-amoxiclav does not change the conformation of HSA at physiological temperature up to 42^o^C, however, it has a mild effect on the HSA conformation at 42^o^C. Therefore, the function of HSA could be changed under these conditions. Moreover, raising the concentration of drug up to 3 folds that of the normal dosage, shows that the optical properties of HSA are almost the same as its optical properties in the absence of drug. 

Interestingly, temperature also affects the denaturation conditions of HSA in the presence of drug. For instance, the denaturation process begins from 10 folds that of the normal dosage of co-amoxiclav at 37^o^C; however, it shifts to 4 folds the normal dosage of drug at 42^o^C. It could be reported that the conformation of HSA is more sensitive to denaturation at high concentrations of drug in the pathological temperature. 

**Figure 3:**
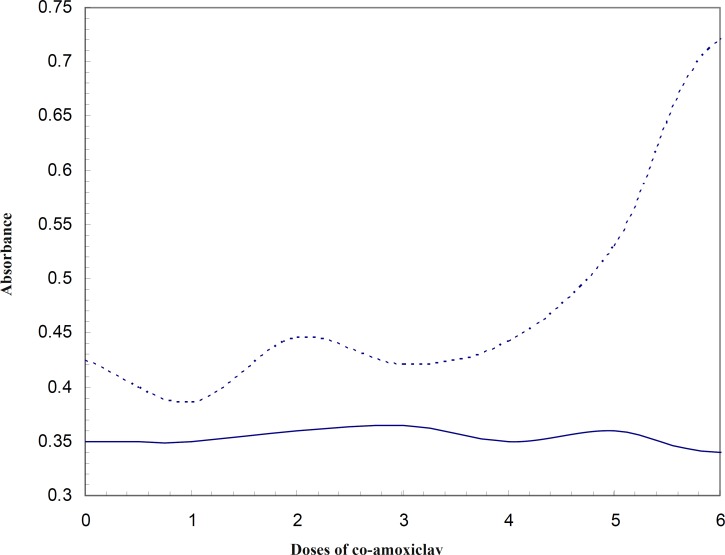
Asorbance of HSA in 280 nm in the presence of different concentrations of co-amoxiclav (Zero represents the absorbance of drug and dosage 1 represents therapeutic dose; 2 represents a dose 2 folds that of the therapeutic dose,…), — line and ----- line represent 37^o^C and 42^o^C, respectively

Comparing the absorbance of HSA at 280 nm at 37^o^C and 42^o^C (Figure 3), reveals that conformational changes of protein due to low doses of co-amoxiclav (from therapeutic up to 6 folds) appears at 42^o^C. This would mean that HSA is more sensitive to conformational changes due to co-amoxiclav in the fever condition and it could affect its binding properties and its affinity to bind the drug and in general its efficiency for transporting. It was reported that HSA has two distinct conformations at 37^o^C and 42^o^C (18). Hence, these two different conformations were compared in terms of their ligand binding properties. Obviously, each of these two conformations is predominant in it’s corresponding temperature.

**Figure 4:**
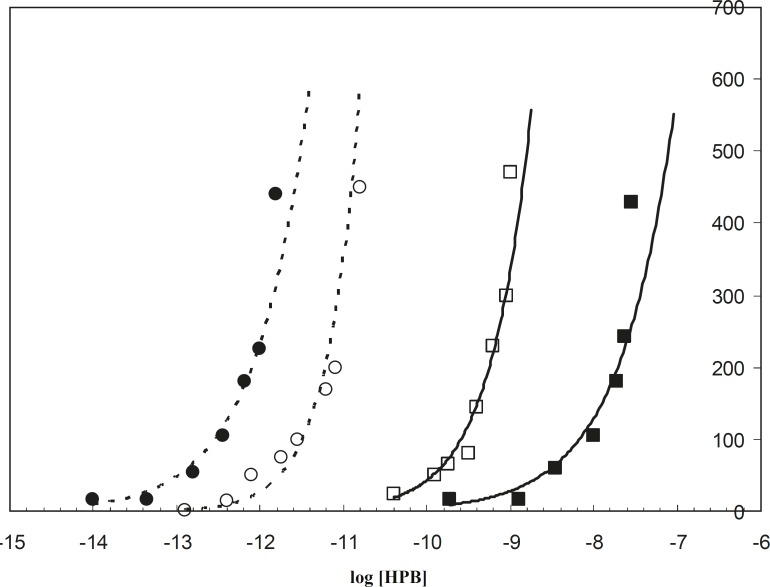
Binding isotherms of HSA – HPB in the absence and presence of half the therapeutical dose of co-amoxiclav. --○-- and --●-- represent respectively the absence and presence of drug at 37^o^C. -□- and -■- represent respectively the absence and presence of drug at 42^o^C

The electrochemistry technique is a well-applied method that has been widely used to study the HSA binding properties to different ligands such as salicylic acid (20) and TiO_2_ (17). In this study the binding of HPB ligand to albumin was carried out in the absence and presence of drug, in order to recognize the alterations of albumin binding properties. Since HSA is saturated in the higher concentrations of HPB at 42^o^C, rather than 37^o^C, it is concluded that HSA has less tendency to HPB in the pathological temperature compared to the physiological temperature. This is in spite of the fact that the binding sites of HSA for ligand (HPB) are the same in both conditions (Figure 4). 

It is known that albumin accounts for 80% of osmotic pressure of plasma (21), from which 30% is due to the presence of HSA itself and 50% is due to the cations (like Na^+^ and K^+^) associated with the HSA negative surface. Reduction of HSA tendency to HPB (a cationic ligand) at 42^o^C relative to 37^o^C, causes a decrease in the negative charge of protein surface. This phenomenon decreases the effect of HSA in the regulation of osmotic pressure in fever conditions. These findings on the binding properties and charge surface density of HSA in both 37^o^C and 42^o^C confirm the different response of HSA versus drug in the mentioned temperatures, since HSA saturation with positively charged ligand (HPB) in the physiological temperature occurred at lower concentrations of ligand (the curve is located on the left side of 42^o^C saturation curve in Figure 4). It could be concluded that HAS surface charge density at 37^o^C is greater than its surface charge density at 42^o^C.

The spectroscopic findings showed different effects of co-amoxiclav on the HSA at 37^o^C and 42^o^C. As shown in Figure 4, HSA in the presence of co-amoxiclav (half the therapeutical dose), is saturated by lower concentrations of HPB at 37^o^C (left shift of the binding isotherm curve) relative to the absence of drug. However, this effect is in contrary to that of 42^o^C. This effect of co-amoxiclav is more noticeable for the higher concentrations of drug (data have not been shown). It can be concluded that the difference between the affinity of HSA for cationic reagents (similar to the related drugs) at 37^o^C and 42^o^C is considerable (see Figure 4, for the difference between the two isotherms in the presence of co-amoxiclav). It may also play an important role in determining the accurate therapeutic dosage of these drugs for physiological and pathological temperatures. It seems that HSA conformation and its affinity are sensitive to drugs and fever [the effect of acetaminophen on the HSA conformational changes has been reported previously (19)]. Therefore, consideration of these effects for determining the accurate dosage of drugs under certain conditions would be compulsory. *In vivo *studies to clarify other aspects of these effects are suggested.

In conclusion, normal doses of co-amoxiclav has no effect on the HSA tertiary structure at 37^o^C, however it has a mild effect on the HSA optical properties at 42^o^C. In another word, HSA is more sensitive to the conformational changes due to co-amoxiclav in the fever condition and it could affect its binding properties and its affinity to bind the drug. In addition, charge density of the HSA surface of is decreased at 42^o^C compared to 37^o^C. Hence, this effect leads to a decrease in the role of HSA in the regulation of osmotic pressure in the fever conditions rather than the physiological conditions. Co-amoxiclav reduces the charge surface density of HSA at physiological and pathological temperatures and therefore alters its binding properties, which could be important in drug interference and complications. 
